# Supplementary material for: NUDT15 polymorphism influences the metabolism and therapeutic effects of acyclovir and ganciclovir
Source: Nat Commun. 2021 Jul 7;12:4181. doi: 10.1038/s41467-021-24509-7 (PMC8263746; doi:10.1038/s41467-021-24509-7)
Supplement: Supplementary file 1 — Supplementary Information [file 41467_2021_24509_MOESM1_ESM.pdf]

Supplementary information

NUDT15 polymorphism influences the metabolism and therapeutic effects of acyclovir and ganciclovir

Variable	*n*
Age at HSCT (years)	36.3 (0 – 70)*
Gender	
male	135 (54.4%)
female	113 (45.6%)
Ethnicity	
Japanese	245 (98.8%)
other	3 (1.2%)
Malignant disease	
Yes	215 (86.7%)
No	33 (13.3%)
CMV seropositivity (donor/recipient)	
negative/negative	39 (15.7%)
positive/negative	69 (27.8%)
negative/positive	36 (14.5%)
positive/positive	103 (41.6%)
Unknown	1 (0.4%)
Donor type	
bone marrow	234 (94.4%)
peripheral blood	3 (1.2%)
cord blood	11 (4.4%)
Conditioning regimen	
myeloablative	132 (53.2%)
non-myeloablative	116 (46.8%)

Supplementary Table 1. **Demographic and clinical characteristics of hematopoietic stem cell transplant patients.**
*One patient without age information was excluded.
HSCT: hematopoietic stem cell transplantation, CMV: cytomegalovirus

	NUDT15 + acyclovir monophosphate<br>PDB ID: 7B7V
Data Collection	
Beamline	SLS PXI
Wavelength (Å)	1.00
Space group	$P\ 2_1\ 2_1\ 2_1$
Cell dimensions	
a, *b*, *c* (Å)	46.64, 49.12, 135.22
α, β, γ (°)	90, 90, 90
Resolution (Å)	44.09 - 1.60 (1.63 - 1.60) *
R_{merge}	5.9 (84.9)*
CC1/2	0.998 (0.639)*
$\langle I / \sigma I \rangle$	12.5 (1.0)*
Total observations	294,062 (8,568)*
Unique observations	41,732 (2,014)*
Completeness (%)	99.7 (98.9)*
Redundancy	7.1 (4.3)*
Refinement	
R_{work} / R_{free}	18.7 / 20.9
No. atoms	
Protein	2,568
Ligand / ion	46
Water	233
Average *B*-factors (Å^2)	
Protein	34.3
Ligand / ion	35.4
Water	39.4
R.m.s. deviations	
Bond lengths (Å)	0.013
Bond angles (°)	1.45
Ramachandran statistics	
Favoured (%)	98
Outliers (%)	0

A single crystal was used for data collection. *Values in parentheses are for highest-resolution shell.

Supplementary Table 2. **Data collection and refinement statistics of NUDT15 crystallography with ACV-MP.**

Gene	Sequence (5′-3′)
IE1	
Forward	TCAGCCATCAACTCTGCTACCAAC
Reverse	ATCTGAAACAGCCGTATATCATCTTG
Probe	56-FAM/TTCTCTGTCAGCTAGCCAATGATATCTTCGAGC/36-TAMSp
HSV-TK	
Forward	TACCCGAGCCGATGACTTA
Reverse	CGGTGTTGTGTGGTGTAGAT
18S	
Forward	TGTGCCGCTAGAGGTGAAATT
Reverse	TGGCAAATGCTTTCGCTTT

Supplementary Table 3. **qPCR primer sequences.**

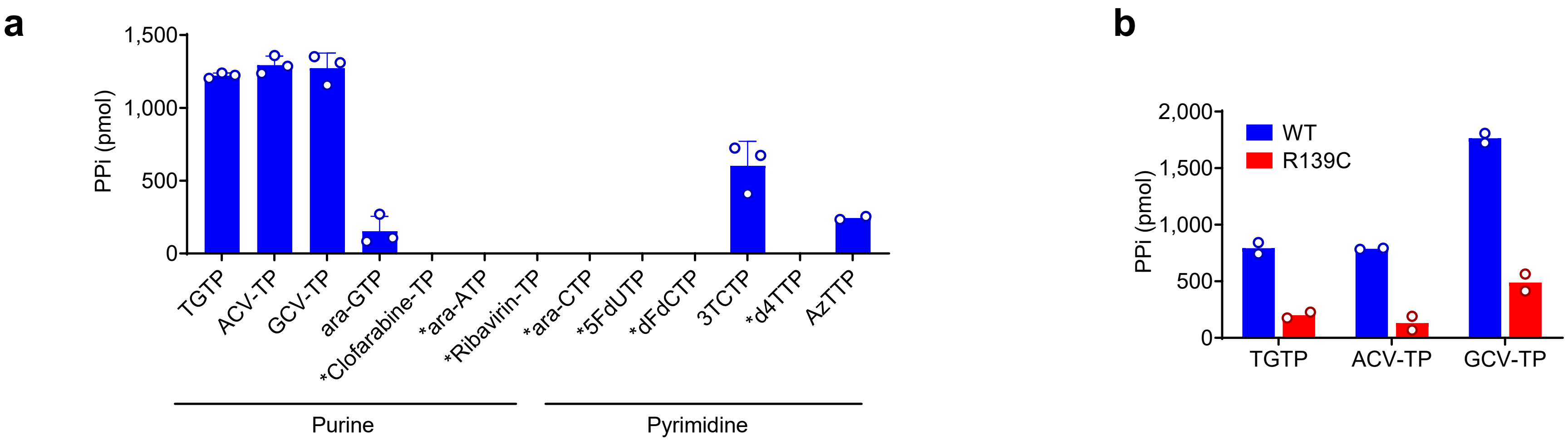

Supplementary Figure 1. **NUDT15 diphosphatase activity on nucleotide analogs. a** Purified NUDT15 wildtype (WT) protein (200 ng) was incubated with various nucleotide analogs for 2 hours followed by measuring released pyrophosphate (PPi) to evaluate the diphosphatase activity. Asterisks denote the value was below the detection limit. Data represent the mean of three replicates; error bars, s.d. **b** NUDT15 diphosphatase activity was compared among different substrates, thioguanosine triphosphate (TGTP), acyclovir triphosphate (ACV-TP) and ganciclovir-triphosphate (GCV-TP). NUDT15 WT or p. R139C protein (100 ng) was incubated with 25 μM substrates, and PPi amount in reaction samples was measured. Data represent the mean of duplicates and experiments were repeated three times. Source data are provided as a Source Data file.

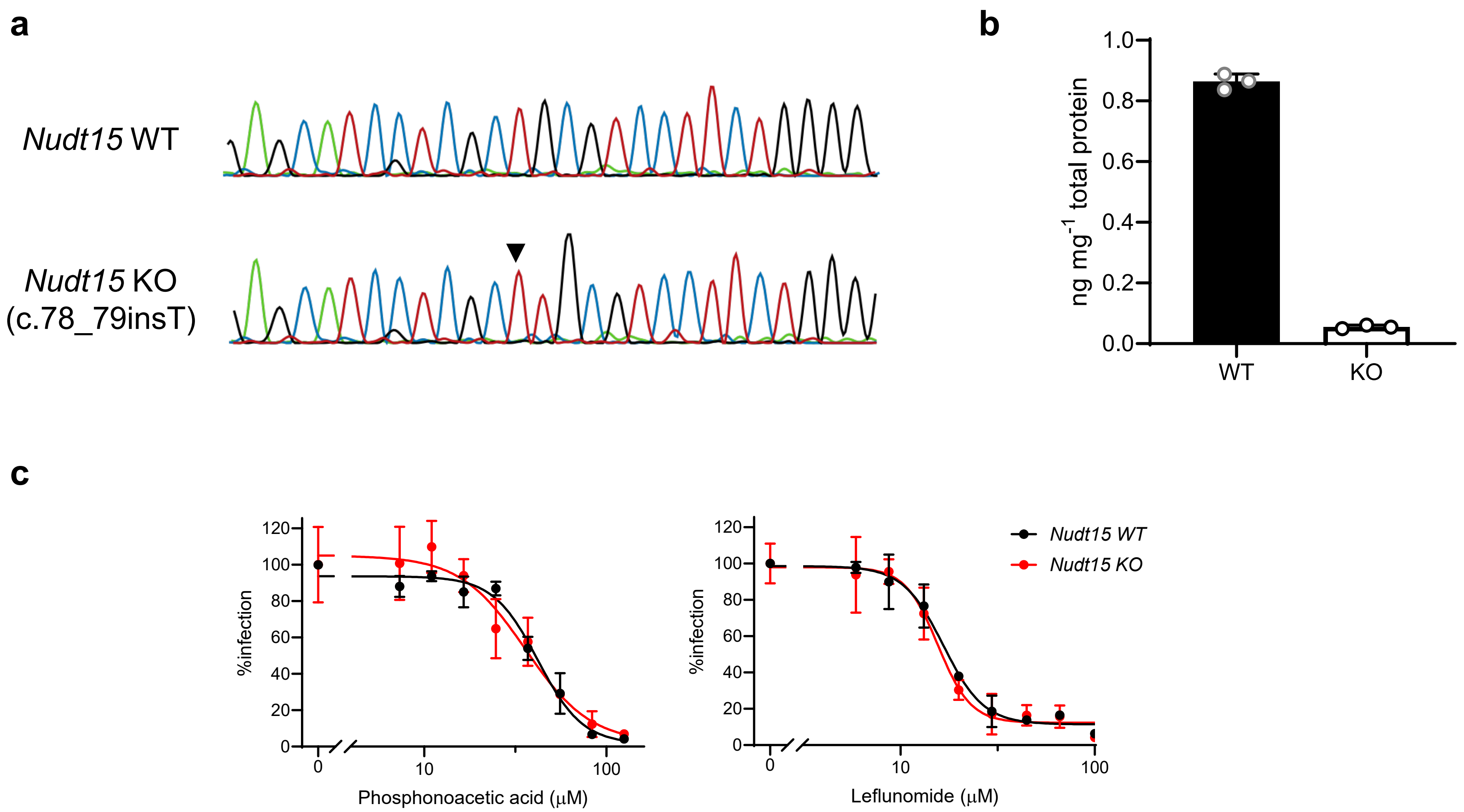

Supplementary Figure 2. **Confirmation of *Nudt15* knockout in mouse M2-10B4 cells.** Endogenous *Nudt15* in M2-10B4 cells was knocked out using the CRISPR-Cas9 technique. A single clone harboring homozygous c. 78_79insT in *Nudt15* was isolated from an edited pool and used for further experiments. **a** A single base insertion of c. 78_79insT was confirmed by Sanger sequencing. **b** The loss of NUDT15 protein was confirmed by sandwich ELISA with monoclonal antibodies generated in-house. The deleterious effects of this insertion on Nudt15 activity was confirmed *in vivo* by us previously (*Blood* 2018 131:2466). **c** No effects were observed on the anti-CMV effects of phosphonoacetic acid and leflunomide. In **b** and **c**, data represent the mean of three replicates; error bars, s.d. Source data for **b** and **c** are provided as a Source Data file.

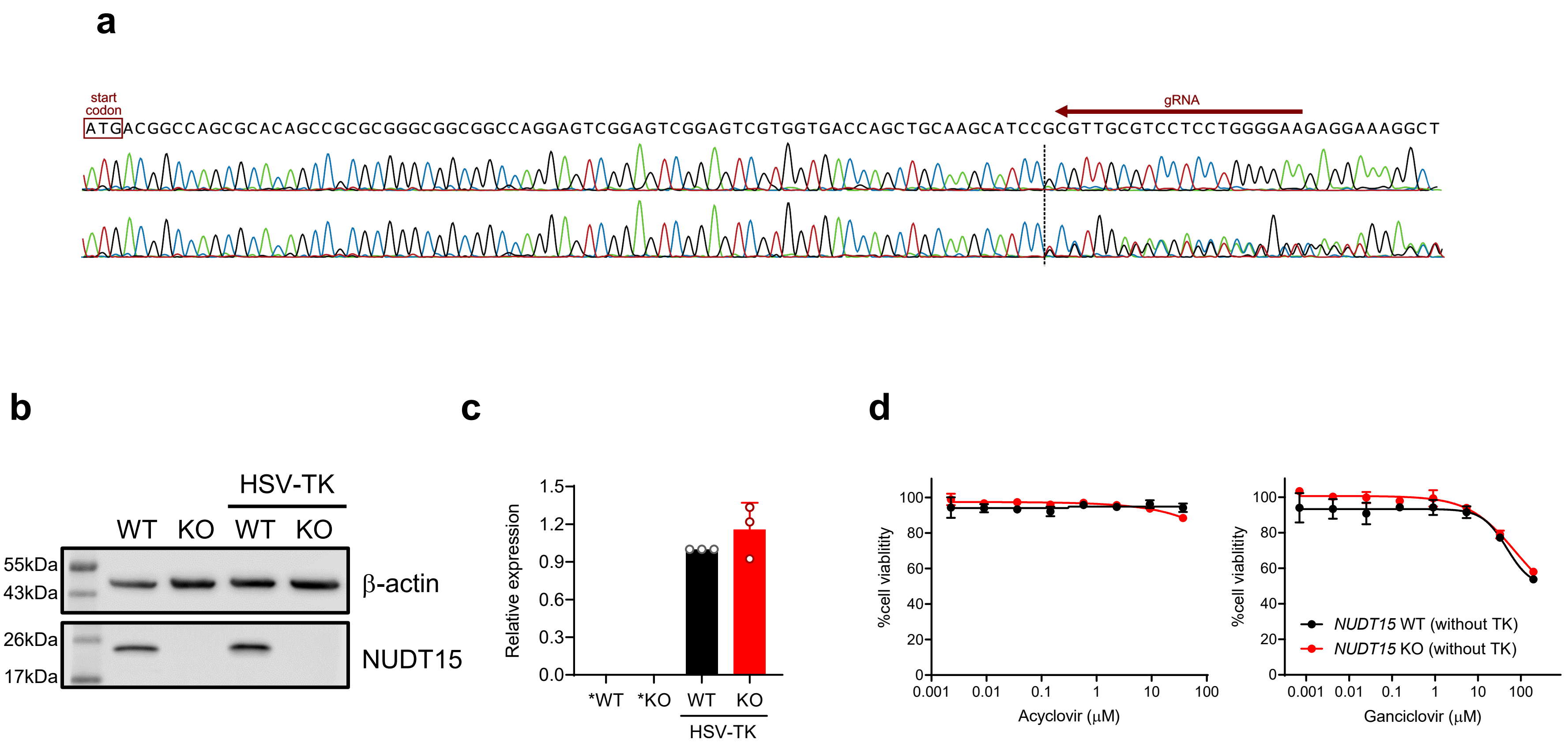

Supplementary Figure 3. ***NUDT15* knockout and ectopic expression of HSV-TK in Nalm6 cells.** Endogenous *NUDT15* in Nalm6 cells was deleted using the CRISPR-Cas9 technique followed by clonal selection. *NUDT15* knockout in a single clone isolated from an edited pool was verified by Sanger sequencing **a** and Western blotting **b**. Representative images from three individual experiments were shown in **b**. **c** *NUDT15* WT and KO cells were transduced with a lentiviral supernatant expressing cl20c-HSV1-TK-IRES-GFP at a low MOI (<0.2). qRT-PCR was performed and there was no significant difference in HSV-TK expression between WT and KO cells. Asterisks denote the value was below the detection limit. Data represent the mean of three individual experiments; error bars, s.d. Student's t-test was performed to compare WT with KO. **d** Cytotoxicity of acyclovir (ACV) and ganciclovir (GCV) in Nalm6 cells without HSV-TK expression was evaluated. Nalm6 cells were largely resistant to ACV and GCV in the absence of HSV-TK. Data represent the mean of three replicates; error bars, s.d. Source data for **b, c** and **d** are provided as a Source Data file.

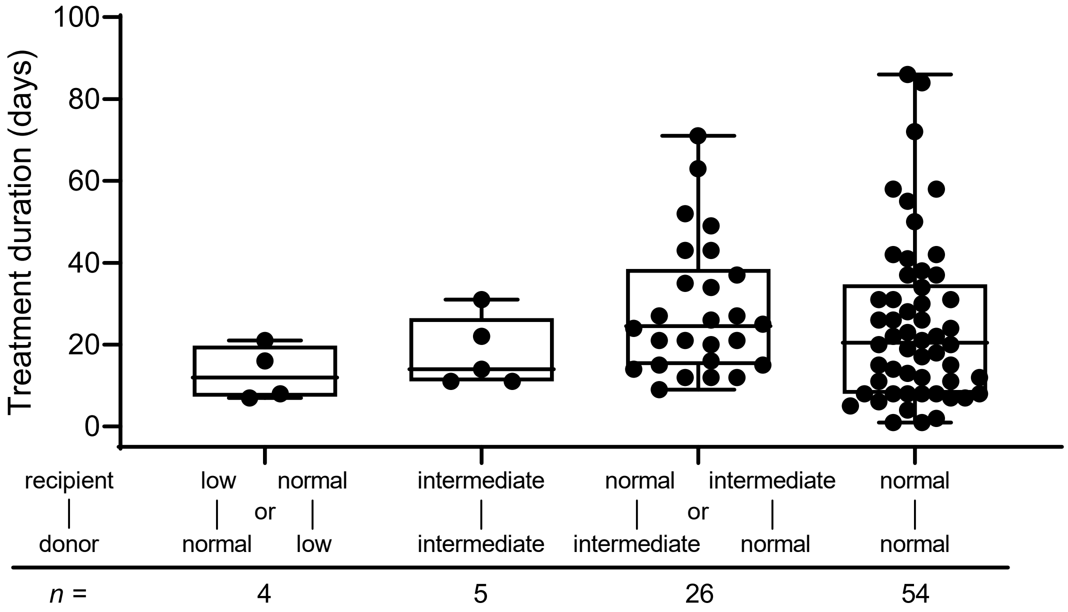

Supplementary Figure 4. **Relationship between *NUDT15* diplotypes and the duration of cytomegalovirus treatment.** In our cohort of hematopoietic stem cell transplant patients, ganciclovir or valganciclovir were initiated upon the detection of CMV viremia. The duration of these CMV treatment was plotted by the combination of *NUDT15* diplotypes in recipient and donor as follows: low/normal or normal/low; intermediate/intermediate; normal/intermediate or intermediate/normal; normal/normal. Although there was a trend for shorter CMV treatment in patients with lower NUDT15 diplotypes, this association did not reach statistical significance. Each box includes data between the 25th and 75th percentiles, with the horizontal line indicating the median and whiskers indicating the maximum and minimum. Source data are provided as a Source Data file.
