# Supplementary material for: NUDT15 polymorphism influences the metabolism and therapeutic effects of acyclovir and ganciclovir
Source: Nat Commun. 2021 Jul 7;12:4181. doi: 10.1038/s41467-021-24509-7 (PMC8263746; doi:10.1038/s41467-021-24509-7)
Supplement: Supplementary file 3 — Reporting Summary [file 41467_2021_24509_MOESM3_ESM.pdf]

nature research

Corresponding author(s): Jun J. Yang

Last updated by author(s): May 9, 2021

Reporting Summary

Nature Research wishes to improve the reproducibility of the work that we publish. This form provides structure for consistency and transparency in reporting. For further information on Nature Research policies, see our Editorial Policies and the Editorial Policy Checklist.

Statistics

For all statistical analyses, confirm that the following items are present in the figure legend, table legend, main text, or Methods section.

n/a	Confirmed	
☐	☒	The exact sample size (*n*) for each experimental group/condition, given as a discrete number and unit of measurement
☐	☒	A statement on whether measurements were taken from distinct samples or whether the same sample was measured repeatedly
☐	☒	The statistical test(s) used AND whether they are one- or two-sided<br>*Only common tests should be described solely by name; describe more complex techniques in the Methods section.*
☐	☒	A description of all covariates tested
☒	☐	A description of any assumptions or corrections, such as tests of normality and adjustment for multiple comparisons
☐	☒	A full description of the statistical parameters including central tendency (e.g. means) or other basic estimates (e.g. regression coefficient) AND variation (e.g. standard deviation) or associated estimates of uncertainty (e.g. confidence intervals)
☐	☒	For null hypothesis testing, the test statistic (e.g. *F*, *t*, *r*) with confidence intervals, effect sizes, degrees of freedom and *P* value noted<br>*Give P values as exact values whenever suitable.*
☒	☐	For Bayesian analysis, information on the choice of priors and Markov chain Monte Carlo settings
☒	☐	For hierarchical and complex designs, identification of the appropriate level for tests and full reporting of outcomes
☒	☐	Estimates of effect sizes (e.g. Cohen's *d*, Pearson's *r*), indicating how they were calculated

Our web collection on statistics for biologists contains articles on many of the points above.

Software and code

Policy information about availability of computer code

Data collection	See below for description of data collection. Full description can be found in the Methods section.<br>Western Blotting - Odyssey Fc Imaging System; Image Studio (LI-COR, version 4.0)<br>MTT, ELISA - BioTek Synergy™ H4<br>IF Imaging - Operetta CLS™ high-content analysis system; Harmony software version 4.9 (Perkin Elmer)<br>UPLC-MS/MS - Acquity UPLC system coupled with ABSciex Trip Quad 6500 system
Data analysis	GraphPad Prism (version 8.3.0 for Windows), R (version 3.6.0), DIALS (version 3.1.0), PHENIX (version 1.18.2, Refine and Phaser are part of the PHENIX suite), Coot (version 0.9), TLSMD (Webserver accessed Oct 4, 2020)

For manuscripts utilizing custom algorithms or software that are central to the research but not yet described in published literature, software must be made available to editors and reviewers. We strongly encourage code deposition in a community repository (e.g. GitHub). See the Nature Research guidelines for submitting code & software for further information.

Data

Policy information about availability of data

All manuscripts must include a data availability statement. This statement should provide the following information, where applicable:

- Accession codes, unique identifiers, or web links for publicly available datasets
- A list of figures that have associated raw data
- A description of any restrictions on data availability

Structural data referenced in this study is available in the Protein Data Bank with the accession code 5LPG [http://doi.org/10.2210/pdb5LPG/pdb]. Structural data generated during the study have been deposited in the Protein Data Bank with the accession code 7B7V [http://doi.org/10.2210/pdb7B7V/pdb]. All other data

supporting the findings of this study are available on reasonable request from the corresponding author. Source data are provided with this paper.

Field-specific reporting

Please select the one below that is the best fit for your research. If you are not sure, read the appropriate sections before making your selection.

- [x] Life sciences
- [ ] Behavioural & social sciences
- [ ] Ecological, evolutionary & environmental sciences

For a reference copy of the document with all sections, see nature.com/documents/nr-reporting-summary-flat.pdf

Life sciences study design

All studies must disclose on these points even when the disclosure is negative.

Sample size	Sample size was determined on the basis of clinical data and sample availability.
Data exclusions	No data was excluded.
Replication	All in vitro experiments have been individually repeated at least three times. All attempts at replication were successful.
Randomization	Randomization is not applicable for in vitro experiment. In the human studies, no randomization was conducted since this study was retrospective and did not include any interventional trials. For the retrospective association study, subjects were selected only based on sample and data availability.
Blinding	Clinical data were used retrospectively. At the time clinical data were collected, investigators had no information about genotype or about this research.

Reporting for specific materials, systems and methods

We require information from authors about some types of materials, experimental systems and methods used in many studies. Here, indicate whether each material, system or method listed is relevant to your study. If you are not sure if a list item applies to your research, read the appropriate section before selecting a response.

Materials & experimental systems

n/a	Involved in the study	
[ ]	[x]	Antibodies
[ ]	[x]	Eukaryotic cell lines
[x]	[ ]	Palaeontology and archaeology
[x]	[ ]	Animals and other organisms
[ ]	[x]	Human research participants
[x]	[ ]	Clinical data
[x]	[ ]	Dual use research of concern

Methods

n/a	Involved in the study	
[x]	[ ]	ChIP-seq
[x]	[ ]	Flow cytometry
[x]	[ ]	MRI-based neuroimaging

Antibodies

Antibodies used	NUDT15 (MYBioSource, MBS2541005), β-actin (Cell Signaling, #4970, 13E5), MCMV m123/IE1 (University of Rijeka, HR-MCMV-08, CROMA101), Anti-mouse IgG Alexa Fluor 488 (Thermo Fisher Scientific, A-11029), NUDT15 (generated in-house, monoclonal, Clone#1-7, #4-10)

Validation	NUDT15 (MYBioSource) - WB and IHC validated for human<br>https://www.mybiosource.com/human-antibody/nudt15/2541005<br><br>β-actin (Cell Signaling, 13E5) - WB, IHC, IF and Flow validated for human, mouse, rat, monkey, bovine and pig<br>https://www.cellsignal.com/products/primary-antibodies/b-actin-13e5-rabbit-mab/4970?site-search-type=Products&N=4294956287&Ntt=13e5&fromPage=plp<br><br>MCMV m123/IE1 (University of Rijeka, CROMA101) - WB, IHC and IF validated for murine cytomegarovirus<br>https://products.capri.com.hr/product/anti-m123-ie1-mcmv-2/<br><br>Anti-mouse IgG Alexa Fluor 488 (Thermo Fisher Scientific) - IHC, ICC, IF and Flow validated for mouse<br>https://www.thermofisher.com/antibody/product/Goat-anti-Mouse-IgG-H-L-Highly-Cross-Adsorbed-Secondary-Antibody-Polyclonal/A-11029<br><br>Nudt15(generated in-house) - ELISA validated<br>We have validated using positivite and negative controls (i.e., Nudt15 overexpressed and knockout cells).

Eukaryotic cell lines

Policy information about cell lines

Cell line source(s)	Nalm6 and M2-10B4 cells were purchased from ATCC.
Authentication	We have done authetification for Nalm6 and M2-10B4 cells by STR analyses.
Mycoplasma contamination	All cell lines have been tested to be mycoplasma negative.
Commonly misidentified lines<br>(See ICLAC register)	No commonly misidentified lines were involved in this study.

Human research participants

Policy information about studies involving human research participants

Population characteristics	Patients involved both children and adults, male and female. Exact details of demographic data of each participant are included in the Supplemental Table 1.
Recruitment	This is a retrospective study and no subjects were specifically recruited for this research. we only utilized cases with sample and data available and those broadly consented to research use of their specimen and data. There is no self-selection bias or other biases that may be present to our knowledge.
Ethics oversight	This project was done under approval of the ethics committee of National Center for Child Health and Development.

Note that full information on the approval of the study protocol must also be provided in the manuscript.
